# Clinical spectrum and features of activated phosphoinositide 3-kinase δ syndrome: A large patient cohort study

**DOI:** 10.1016/j.jaci.2016.06.021

**Published:** 2017-02

**Authors:** Tanya I. Coulter, Anita Chandra, Chris M. Bacon, Judith Babar, James Curtis, Nick Screaton, John R. Goodlad, George Farmer, Cathal Laurence Steele, Timothy Ronan Leahy, Rainer Doffinger, Helen Baxendale, Jolanta Bernatoniene, J. David M. Edgar, Hilary J. Longhurst, Stephan Ehl, Carsten Speckmann, Bodo Grimbacher, Anna Sediva, Tomas Milota, Saul N. Faust, Anthony P. Williams, Grant Hayman, Zeynep Yesim Kucuk, Rosie Hague, Paul French, Richard Brooker, Peter Forsyth, Richard Herriot, Caterina Cancrini, Paolo Palma, Paola Ariganello, Niall Conlon, Conleth Feighery, Patrick J. Gavin, Alison Jones, Kohsuke Imai, Mohammad A.A. Ibrahim, Gašper Markelj, Mario Abinun, Frédéric Rieux-Laucat, Sylvain Latour, Isabelle Pellier, Alain Fischer, Fabien Touzot, Jean-Laurent Casanova, Anne Durandy, Siobhan O. Burns, Sinisa Savic, D.S. Kumararatne, Despina Moshous, Sven Kracker, Bart Vanhaesebroeck, Klaus Okkenhaug, Capucine Picard, Sergey Nejentsev, Alison M. Condliffe, Andrew James Cant

**Affiliations:** a Department of Immunology, School of Medicine, Trinity College, Dublin, and St James's Hospital, Dublin, Ireland; b Department of Paediatric Immunology and Infectious Diseases, Our Lady's Children's Hospital Crumlin, Dublin, Ireland; c Department of Clinical Biochemistry and Immunology, Addenbrooke's Hospital, Cambridge, United Kingdom; d Lymphocyte Signalling & Development, Babraham Institute, Cambridge, United Kingdom; e Department of Medicine, University of Cambridge, Cambridge, United Kingdom; f Northern Institute for Cancer Research, Newcastle University, Newcastle upon Tyne, United Kingdom; g Department of Radiology, Cambridge University Hospitals NHS Foundation Trust, Cambridge, United Kingdom; h Raigmore Hospital, Inverness, United Kingdom; i Regional Immunology Service, The Royal Hospitals, Belfast, United Kingdom; j National Institute for Health Research, Cambridge Biomedical Research Centre, Cambridge, United Kingdom; k Department of Infectious Disease and Immunology, University Hospitals Bristol NHS Foundation Trust, Bristol Royal Hospital for Children, Bristol, United Kingdom; l Barts Health NHS Trust, London, United Kingdom; m Center for Chronic Immunodeficiency, University Hospital Freiburg, Freiburg, Germany; n Department of Pediatrics and Adolescent Medicine, University Medical Center, Freiburg, Germany; o Institute of Immunology, University Hospital Motol, Prague, Czech Republic; p Faculty of Medicine and Institute of Life Sciences, University of Southampton, Southampton, United Kingdom; q NIHR Wellcome Trust Clinical Research Facility, University Hospital Southampton NHS Foundation Trust, Southampton, United Kingdom; r Department of Immunology, Epsom & St Helier University Hospitals NHS Trust, Surrey, United Kingdom; s Division of Bone Marrow Transplantation and Immune Deficiency, Cincinnati Children's Hospital Medical Center, Cincinnati, Ohio; t Royal Aberdeen Childrens' Hospital, Aberdeen, United Kingdom; u Department of Pediatrics, Ospedale Pediatrico Bambino Gesù and University of Rome “Tor Vergata”, Rome, Italy; v Department of Immunology, Great Ormond Street Hospital NHS Foundation Trust, London, United Kingdom; w King's College London, King's Health Partners, King's College Hospital NHS Foundation Trust, School of Medicine, Division of Asthma, Allergy & Lung Biology, Department of Immunological Medicine, London, United Kingdom; x Department of Allergology, Rheumatology and Clinical Immunology, University Children's Hospital, University Medical Center, Ljubljana, Slovenia; y Department of Paediatric Immunology, Newcastle upon Tyne hospitals NHS Foundation Trust, Newcastle upon Tyne, United Kingdom; z Départment de Biothérapie, Centre d'Investigation Clinique intégré en Biothérapies, Necker Children's Hospital, Assistance Publique-Hôpitaux de Paris (AP-HP), Paris, France; aa Université Paris Descartes–Sorbonne Paris Cité, Institut Imagine, Paris, France; bb INSERM UMR1163, Paris, France; cc Department of Pediatric Immunology, Hematology and Rheumatology, AP-HP, Necker Children's Hospital, Paris, France; dd Unité d'Onco-hémato-immunologie Pédiatrique, CHU Angers, Angers, France; ee Centre de Référence Déficits Immunitaires Héréditaires, AP-HP, Paris, France; ff Inserm UMR 892, Angers, France; gg CNRS UMR 6299, Angers, France; hh Collège de France, Paris, France; ii Laboratory of Human Genetics of Infectious Diseases, Necker Branch, INSERM UMR1163, Imagine Institute, Necker Children's Hospital, Paris, France; jj St Giles Laboratory of Human Genetics of Infectious Diseases, Rockefeller Branch, Rockefeller University, New York, NY; kk Howard Hughes Medical Institute, Chevy Chase, Md; ll University College London Institute of Immunity and Transplantation, London, United Kingdom; mm Department of Clinical Immunology and Allergy, St James's University Hospital, Leeds, United Kingdom; nn UCL Cancer Institute, University College London, London, United Kingdom; oo Department of Infection, Immunity and Cardiovascular Disease, University of Sheffield, Sheffield, United Kingdom; pp Institute of Cellular Medicine, Newcastle University, Newcastle upon Tyne Hospitals NHS Trust, Newcastle upon Tyne, United Kingdom; qq Northern England Haemato-Oncology Diagnostic Service, Newcastle upon Tyne NHS Foundation Trust, Newcastle upon Tyne, United Kingdom; rr Papworth Hospital NHS trust, Papworth Everard, Cambridge, United Kingdom; ss Department of Radiology, Papworth Hospital NHS Foundation Trust, Papworth Everard Hospital, Cambridge, United Kingdom; tt Department of Pathology, Western General Hospital, Edinburgh, United Kingdom; uu Department of Royal Hospital for Children, Glasgow, United Kingdom; vv Department of Pathology, Queen Elizabeth University Hospital, Glasgow, United Kingdom; ww Department of Community Pediatrics, Perinatal and Maternal Medicine Tokyo Medical and Dental University (TMDU), Tokyo, Japan

**Keywords:** Activated phosphoinositide 3-kinase δ syndrome, p110δ-activating mutation causing senescent T cells, lymphadenopathy, and immunodeficiency, phosphoinositide 3-kinase δ, *PIK3CD* gene, bronchiectasis, immunodeficiency, hematopoietic stem cell transplantation, phosphoinositide 3-kinase inhibitor, APDS, Activated phosphoinositide-3 kinase δ syndrome, BALF, Bronchoalveolar lavage fluid, CMV, Cytomegalovirus, CNS, Central nervous system, CT, Computed tomography, GOF, Gain of function, HSCT, Hematopoietic stem cell transplantation, HSV, Herpes simplex virus, OR, Odds ratio, PI3K, Phosphoinositide 3-kinase, PPV, Pneumococcal polysaccharide vaccine

## Abstract

**Background:**

Activated phosphoinositide 3-kinase δ syndrome (APDS) is a recently described combined immunodeficiency resulting from gain-of-function mutations in *PIK3CD*, the gene encoding the catalytic subunit of phosphoinositide 3-kinase δ (PI3Kδ).

**Objective:**

We sought to review the clinical, immunologic, histopathologic, and radiologic features of APDS in a large genetically defined international cohort.

**Methods:**

We applied a clinical questionnaire and performed review of medical notes, radiology, histopathology, and laboratory investigations of 53 patients with APDS.

**Results:**

Recurrent sinopulmonary infections (98%) and nonneoplastic lymphoproliferation (75%) were common, often from childhood. Other significant complications included herpesvirus infections (49%), autoinflammatory disease (34%), and lymphoma (13%). Unexpectedly, neurodevelopmental delay occurred in 19% of the cohort, suggesting a role for PI3Kδ in the central nervous system; consistent with this, PI3Kδ is broadly expressed in the developing murine central nervous system. Thoracic imaging revealed high rates of mosaic attenuation (90%) and bronchiectasis (60%). Increased IgM levels (78%), IgG deficiency (43%), and CD4 lymphopenia (84%) were significant immunologic features. No immunologic marker reliably predicted clinical severity, which ranged from asymptomatic to death in early childhood. The majority of patients received immunoglobulin replacement and antibiotic prophylaxis, and 5 patients underwent hematopoietic stem cell transplantation. Five patients died from complications of APDS.

**Conclusion:**

APDS is a combined immunodeficiency with multiple clinical manifestations, many with incomplete penetrance and others with variable expressivity. The severity of complications in some patients supports consideration of hematopoietic stem cell transplantation for severe childhood disease. Clinical trials of selective PI3Kδ inhibitors offer new prospects for APDS treatment.

*Discuss this article on the JACI Journal Club blog:* *www.jaci-online.blogspot.com*.

Activated phosphoinositide 3-kinase δ syndrome (APDS) is an autosomal dominant primary immunodeficiency caused by gain-of-function (GOF) mutations in *PIK3CD*,^1^, ^2^ which encodes the p110δ catalytic subunit of phosphoinositide 3-kinase δ (PI3Kδ). PI3Kδ, a class 1 PI3K isoform generating phosphatidylinositol 3,4,5-trisphosphate, is a heterodimer comprising p110δ and a p85 family regulatory subunit. PI3Kδ is expressed predominantly in leukocytes and plays an important role in their proliferation, survival, and activation.^3^, ^4^, ^5^

Recently, we described 17 patients with a combined immunodeficiency disorder caused by the heterozygous *PIK3CD* GOF mutation E1021K.^1^ Patients' lymphocytes displayed increased basal and poststimulation phosphatidylinositol 3,4,5-trisphosphate and enhanced downstream Akt–mammalian/mechanistic target of rapamycin signaling. This disorder was named APDS.^1^ Lucas et al^2^ independently reported 14 patients with a similar disease caused by E1021K and 2 other activating mutations in *PIK3CD*, designating it p110δ-activating mutation causing senescent T cells, lymphadenopathy, and immunodeficiency (PASLI).^2^ To date, 4 heterozygous GOF *PIK3CD* mutations (E1021K, N334K, E525K, and C416R) have been described, with E1021K the most common.^1^, ^2^, ^6^, ^7^, ^8^ Patients in both cohorts experienced recurrent respiratory tract infections, bronchiectasis, herpesvirus infections, nonneoplastic lymphoproliferation, and lymphoma. However, possibly because of different case-finding strategies, we reported bronchiectasis in 75% of our cohort and herpesvirus infections in 24%, whereas Lucas et al^2^ described bronchiectasis in 33%, but all patients had herpesvirus viremia. Recent reports have also underscored that patients with APDS have a high incidence of lymphoma^7^, ^8^ and possible autoimmune manifestations.^2^, ^9^

In this study we describe the clinical, radiologic, histopathologic, and immunologic features of APDS in a genetically confirmed cohort of 53 patients, the largest to date. We demonstrated a wide spectrum of clinical findings and complications and unexpectedly noted an increased frequency of neurodevelopmental manifestations. These findings will aid clinical decision making in the diagnosis and treatment of APDS and facilitate patient counseling.

## Methods

Informed consent was obtained from patients, parents, or both. The study conformed to the Declaration of Helsinki and all local ethical requirements.

Mutations in *PIK3CD* were identified by means of Sanger sequencing.^1^ Only patients heterozygous for an APDS-associated GOF *PIK3CD* mutation were included. Twenty-five patients from this cohort have been included in previous reports,^1^, ^7^ and 28 are reported for the first time.

Information on demographics, presentation, complications, laboratory parameters, management, and outcomes was compiled retrospectively by using patient/parent interview and medical note review. Pneumonia and bronchiectasis required radiologic confirmation. Chest computed tomographic (CT) scans from 31 patients were independently reviewed by 2 thoracic radiologists (J.B. and N.S.) for air-space opacity, atelectasis, nodules, bronchiectasis, mosaic attenuation, and lymphadenopathy.^10^, ^11^ Available histopathology specimens (29 specimens from 11 patients) were reviewed by 2 hematopathologists (C.M.B. and J.R.G.). Patients’ most recent immunology results are described; postrituximab B-cell levels were excluded. All laboratory results were analyzed with reference to age-related normal ranges.^12^, ^13^, ^14^, ^15^ A poor pneumococcal polysaccharide vaccine (PPV) response was defined as a less than 4-fold increase in antipneumococcal IgG titer at 4 to 6 weeks after PPV vaccination.

Significant associations in clinical complications were determined by odds ratios (ORs) with 95% CIs and Fisher exact tests by using GraphPad Prism software (version 6; GraphPad Software, La Jolla, Calif). *P* values of less than .05 were considered significant.

## Results

### Patients' characteristics

Fifty-three patients with APDS (34 male patients) from 30 unrelated families were included; 5 patients (4 male) were deceased. Living patients had a mean age of 17.2 years (age range, <1-65 years). Forty-two patients were of European descent, 4 were Afro-Caribbean, 3 were Middle Eastern, 2 were Indian, 1 was Chinese, and 1 was Japanese. Fifty patients were heterozygous for E1021K, and 3 related subjects were heterozygous for E525K.

### Presentation

Recurrent respiratory tract infections occurred in 96% of patients, with onset from less than 1 to 7 years of age. Lymphadenopathy, hepatosplenomegaly, or both were common at presentation (42%). Five patients were identified in adulthood after their child received a diagnosis of APDS; 2 had bronchiectasis and recurrent respiratory tract infections, 1 experienced recurrent respiratory tract infections in childhood and a persistent granulomatous local skin reaction to BCG vaccination, 1 was under investigation for chronic cervical lymphadenopathy, and 1 had no reported health issues. The 4 symptomatic adults had abnormal immunoglobulin profiles, including increased IgM and reduced IgG_2_ levels, although none had a low total IgG level.

### Infective complications

**Fig 1:**
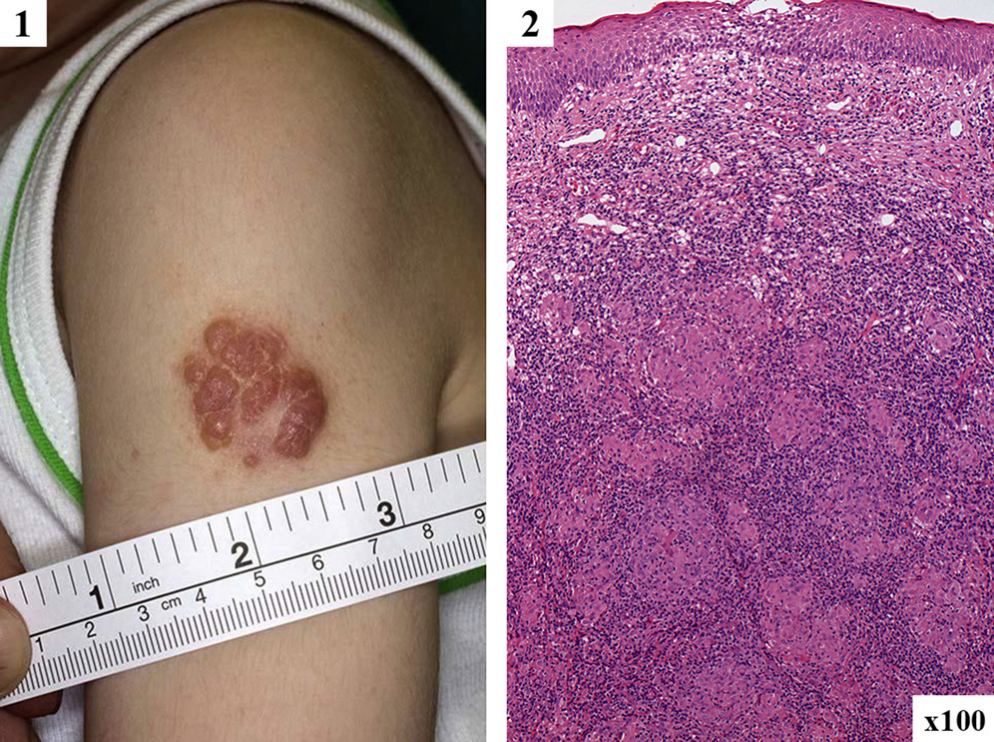
BCG-induced granulomatous inflammation in patients with APDS. *1*, Granulomatous skin lesion in a 4-year-old at the site of BCG vaccination administered at 4 months of age. *2*, Skin biopsy specimen showing granulomatous inflammation.

Pneumonia (85%), bronchiectasis (60%), and upper respiratory tract infections were common, often with childhood onset (Table I). Only 2 patients did not report recurrent respiratory tract infections. The most common bacterial pathogens were *Streptococcus pneumoniae* and *Haemophilus influenzae*, with *Staphylococcus aureus*, *Moraxella catarrhalis*, *Pseudomonas aeruginosa*, and *Klebsiella* species also observed. The mean age at diagnosis of bronchiectasis was 8.6 years (range, 1.3-36 years). Four patients had permanent hearing loss from recurrent otitis media. Non–respiratory tract bacterial infections included ocular infections (21%: conjunctivitis [n = 8], dacryocystitis [n = 3], and orbital cellulitis [n = 2]) and abscesses (17%: *S aureus* skin abscesses [n = 4], salivary gland abscesses [n = 3], dental abscesses [n = 3], and *S pneumoniae* lymph node abscess [n = 1]). No invasive bacterial infections were reported. Two unrelated patients had persistent granulomatous skin lesions at BCG vaccination injection sites (Fig 1); material from 1 lesion was culture positive for BCG. No other mycobacterial infections were reported.

Persistent, severe, or recurrent herpesvirus infections occurred in 49% of patients. EBV viremia was detected in 26%, with 6 (11%) patients having disseminated infection, including 1 case of EBV encephalitis. EBV was detected in lymph node (n = 3), tonsillar (n = 1), palatal (n = 1), and gastrointestinal (n = 1) biopsy specimens, as well as cerebrospinal fluid (n = 1) and bronchoalveolar lavage fluid (BALF; n = 1). Two patients had EBV-positive lymphoma. Eight patients had cytomegalovirus (CMV) viremia, 4 with systemic CMV infection successfully treated with ganciclovir. Four cases of EBV/CMV coinfection occurred. One patient with diffuse lymphadenopathy and hepatosplenomegaly had EBV, CMV, and human herpesvirus 6 identified by using PCR on lymph node biopsy. Two patients were hospitalized with severe primary varicella zoster virus infection, and 2 had recurrent shingles. A nongenotyped sibling reportedly died of varicella zoster virus pneumonitis at age 11 years. Recurrent herpes simplex virus (HSV) infections included oral ulceration (n = 4), skin infections (n = 2), and herpetic keratitis (n = 1). HSV was identified in BALF of 2 symptomatic patients, 1 with severe pneumonitis. Adenovirus infections were reported in 9 (17%) patients, with positive isolates from blood, BALF, and stool. Warts (n = 4) and *Molluscum contangiosum* (n = 4) were extensive in those affected.

*Cryptosporidium parvum* was isolated from a patient with bloody diarrhea at age 6 to 18 months in whom cirrhosis was identified at age 8 years; the liver biopsy specimen was negative for *Cryptosporidium* species. A second patient had *C parvum-*positive diarrhea immediately after hematopoietic stem cell transplantation (HSCT). The only other parasitic infection identified was toxoplasmosis in a 9-month-old child. Oral mucocutaneous candidiasis requiring treatment was reported in 7 (13%) patients, including candida tracheitis (n = 1) and esophageal candidiasis (n = 1). No cases of *Aspergillus* species infection were identified.

### Noninfective immune complications

#### Nonneoplastic lymphoproliferation

Chronic lymphadenopathy, splenomegaly, and/or hepatomegaly were observed in 75% of patients (Table I). Lymphadenopathy typically began in childhood, was persistent or recurrent, and was often localized to sites of infection. There were 14 cases of cervical lymphadenopathy; 8 of 10 patients with persistent intrathoracic lymphadenopathy had bronchiectasis and recurrent consolidation. Seven patients had diffuse lymphadenopathy, and EBV, CMV, or dual viremia was diagnosed in all 6 of these patients in whom viral PCR was performed. Lymphadenopathy was significantly associated with mucosal lymphoid hyperplasia (OR, 16; 95% CI, 1.9-133.8; *P* = .002), splenomegaly (OR, 9.1; 95% CI, 2.5-33.2; *P* = .0005), and herpesvirus infection (OR, 6.9; 95% CI, 1.9-25.2; *P* = .004).

**Fig 2:**
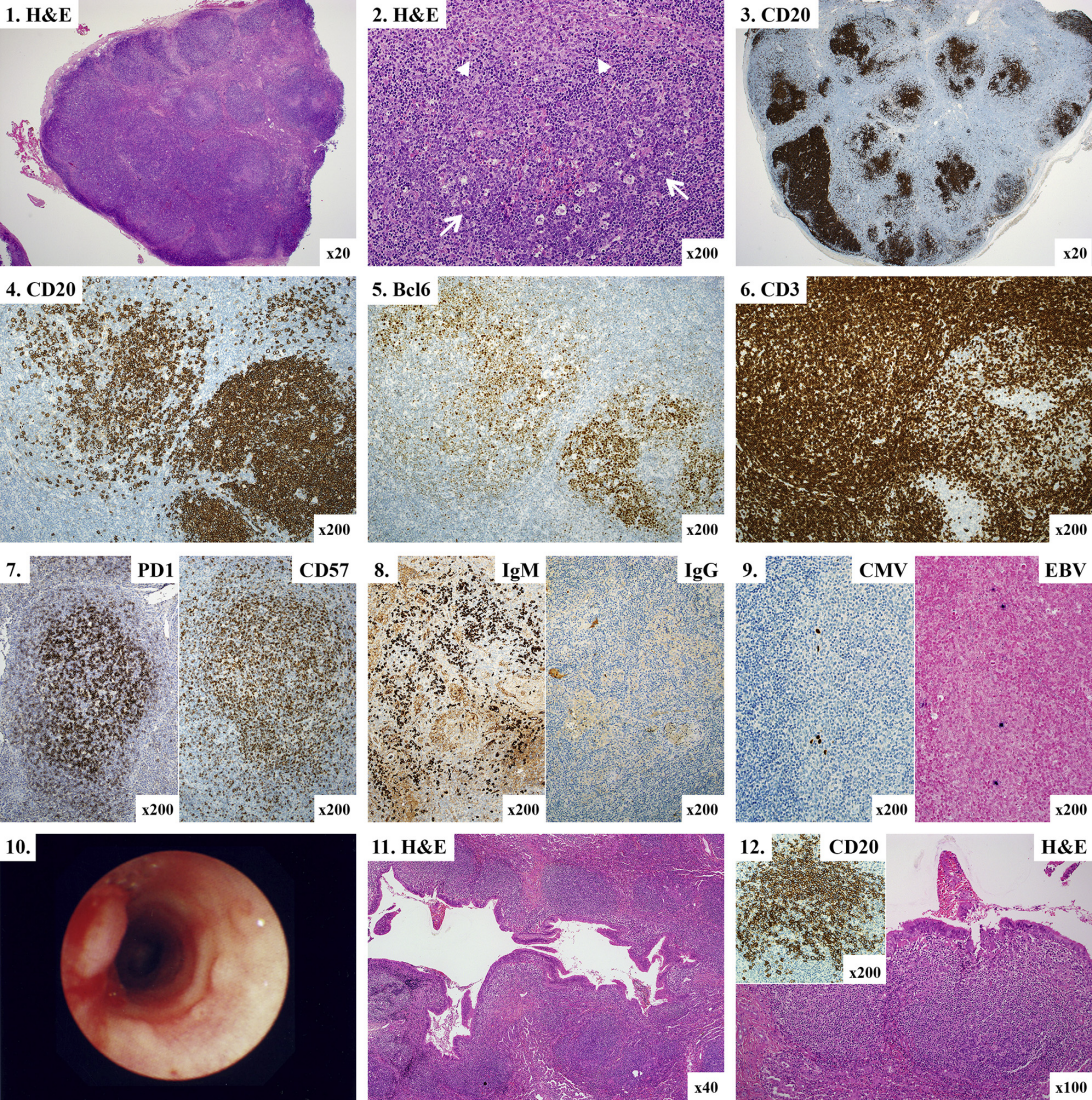
Lymphoid hyperplasia. *1* and *2*, Lymph node showing atypical follicular hyperplasia with disrupted follicles *(arrows)* and monocytoid B cells *(arrowheads)*. *3-5*, Disrupted germinal centers were highlighted by staining for CD20 (Fig 2, *3* and *4*) and Bcl6 (Fig 2, *5*). *6* and *7*, Follicles were infiltrated by T cells (Fig 2, *6*), many of which expressed PD1, CD57, or both (Fig 2, *7*). *8*, IgM-positive plasma cells were present, but IgG-positive plasma cells were reduced or absent. *9*, Several lymph nodes contained CMV or EBV (EBER). *10*, Tracheal endoscopy showing mucosal nodules. *11* and *12*, Lung showing peribronchiolar lymphoid hyperplasia (Fig 2, *11*) with disrupted follicles (Fig 2, *12*). *H&E*, Hematoxylin and eosin.

Histologically (Fig 2), lymph nodes showed atypical follicular hyperplasia with absent or attenuated follicular mantle zones. Germinal centers were frequently disrupted and partially effaced by numerous T cells, many of which were programmed cell death protein 1 (PD1)^+^, CD57^+^, or both, which is consistent with follicular T_H_ cells. Parasinusoidal aggregates of monocytoid B cells were a recurrent feature. IgG^+^ plasma cells were reduced in number. One lymph node showed features analogous to those of posttransplantation lymphoproliferative disorder, which is characterized by a polymorphic infiltrate of B cells, T cells, epithelioid macrophages, and light chain–restricted plasma cells; monocytoid B-cell hyperplasia; and equivocal immunoglobulin gene rearrangement assays. There was no progression to lymphoma on prolonged follow-up. Scattered EBV-positive cells, CMV-positive cells, or both were present in several lymph nodes, but florid infectious mononucleosis-like pathology was not encountered. Mucosal nodular lymphoid hyperplasia was visualized as cobblestone-like plaques or polyps in 17 (32%) patients. In the gastrointestinal tract mucosal lymphoid hyperplasia was identified endoscopically anywhere from the epiglottis to the rectum in 14 (26%) subjects and associated with diarrhea, bleeding, and rectal prolapse. Five patients had respiratory mucosal nodular lymphoid hyperplasia identified bronchoscopically (Fig 2). Biopsy specimens from mucosal lymphoid lesions showed follicular hyperplasia, often with features similar to those seen in lymph nodes (Fig 2), and were occasionally PCR positive for herpes viruses (EBV, n = 1; HSV, n = 1).

#### Autoimmune and inflammatory disease

Thirty-four percent of the cohort had clinical features suggestive of autoimmune or inflammatory disease. Cytopenias included Coombs-positive hemolytic anemia (n = 7) and 2 cases of trilineage cytopenia responsive to steroids or rituximab. Glomerulonephritis affected 3 children, necessitating renal transplantation in 2 cases. Renal biopsy specimens showed proliferative, membranoproliferative, and focal and segmental changes. Two patients had exocrine pancreatic insufficiency. Autoantibody-positive thyroid disease was diagnosed in 3 patients in adulthood. Two patients had seronegative arthritis, and 1 had recurrent pericarditis.

Three patients had cirrhosis, of whom 1 also had sclerosing cholangitis in the setting of previous *Cryptosporidium* species–related diarrhea. Sclerosing cholangitis additionally affected a second noncirrhotic patient who had no evidence of *Cryptosporidium* species infection.^9^ Thirteen (25%) patients had chronic diarrhea, 9 of whom had gastrointestinal nodular mucosal lymphoid hyperplasia confirmed on endoscopy.

#### Lymphoma and other malignancy

**Fig E1:**
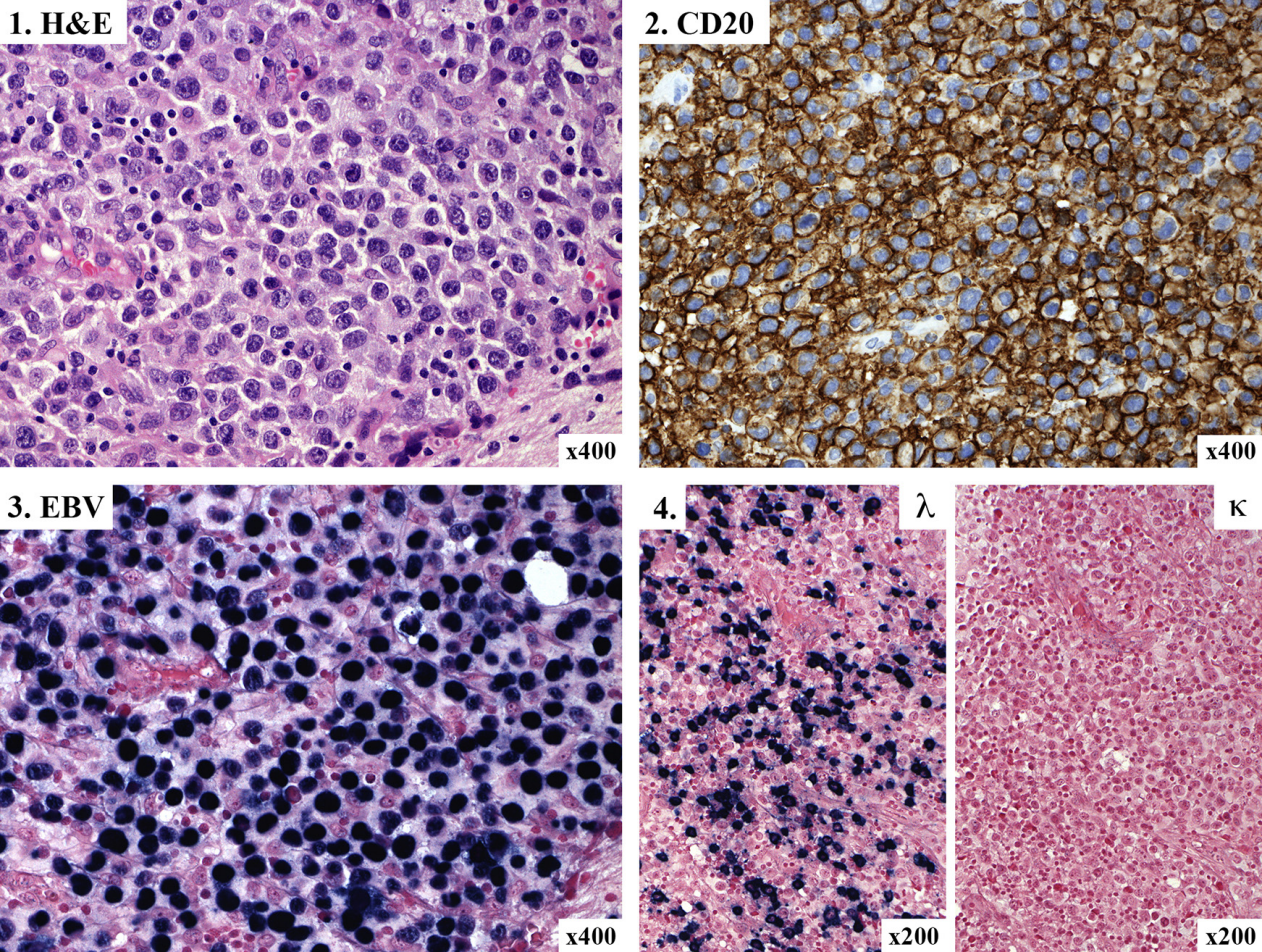
EBV-positive diffuse large B-cell lymphoma in patients with APDS. *1*, A diffuse infiltrate of large atypical lymphoid cells and some atypical plasmacytoid cells was present in the cerebellum. *2*, Immunohistochemical staining showed large B cells expressing CD20, CD79a, Pax5, and interferon regulatory factor 4 but not Bcl6 or CD10. *3*, Most neoplastic cells showed positive *in situ* hybridization for EBV EBER. *4*, Plasmacytoid cells expressed CD138 and showed λ restricted immunoglobulin light chain *in situ* hybridization. *H&E*, Hematoxylin and eosin.

**Fig E2:**
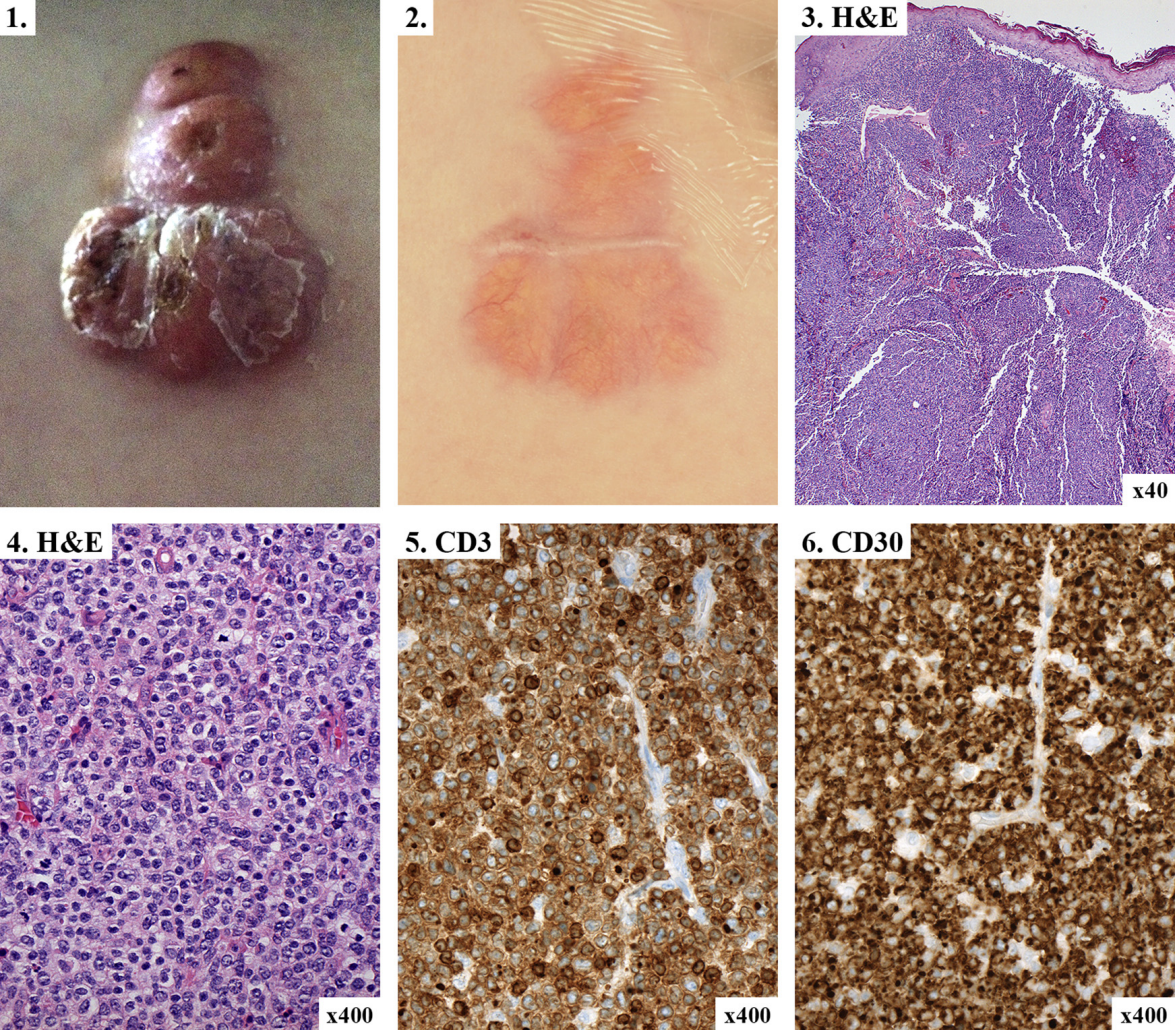
Primary cutaneous anaplastic large cell lymphoma in patients with APDS. *1* and *2*, A multinodular cutaneous tumor on the chest of an 11-year-old boy (Fig E2, *1*), which regressed to a flat plaque (Fig E2, *2*) on 6 weeks of treatment with rapamycin. *3* and *4*, The dermis and subcutis contained a diffuse infiltrate of large atypical lymphoid cells. *5* and *6*, Immunohistochemical staining showed large T cells expressing CD3 (Fig E2, *5*), CD30 (Fig E2, *6*), CD2, interferon regulatory factor 4, T-cell receptor β, and perforin but not CD4, CD8, or ALK. *H&E*, Hematoxylin and eosin.

Seven (13%) patients had lymphoma at age 18 months to 27 years. There were 2 cases of diffuse large B-cell lymphoma, 1 EBV positive (see Fig E1 in this article's Online Repository at www.jacionline.org) and 1 EBV negative.^7^ Single patients were reported as having nodular sclerosis classical Hodgkin lymphoma,^7^ nodal marginal zone lymphoma,^1^ and a lymphoplasmacytic lymphoma, the EBV status of which were unknown. An EBV-positive Hodgkin-type lymphoproliferative disorder was diagnosed in a child after renal transplantation. One child had a primary cutaneous anaplastic large cell lymphoma carrying t(6; 7) (p25; q23). This regressed from a 9 × 6–cm mass of tumor nodules to a 5 × 4–cm diameter flat erythematous plaque on 6 weeks of treatment with rapamycin (sirolimus, see Fig E2 in this article's Online Repository at www.jacionline.org). Three patients died of lymphoma-related complications, including both patients with EBV-associated lymphoma. No other malignancies have been identified within our cohort to date.

#### Neurological and other nonimmune features

**Fig 3:**
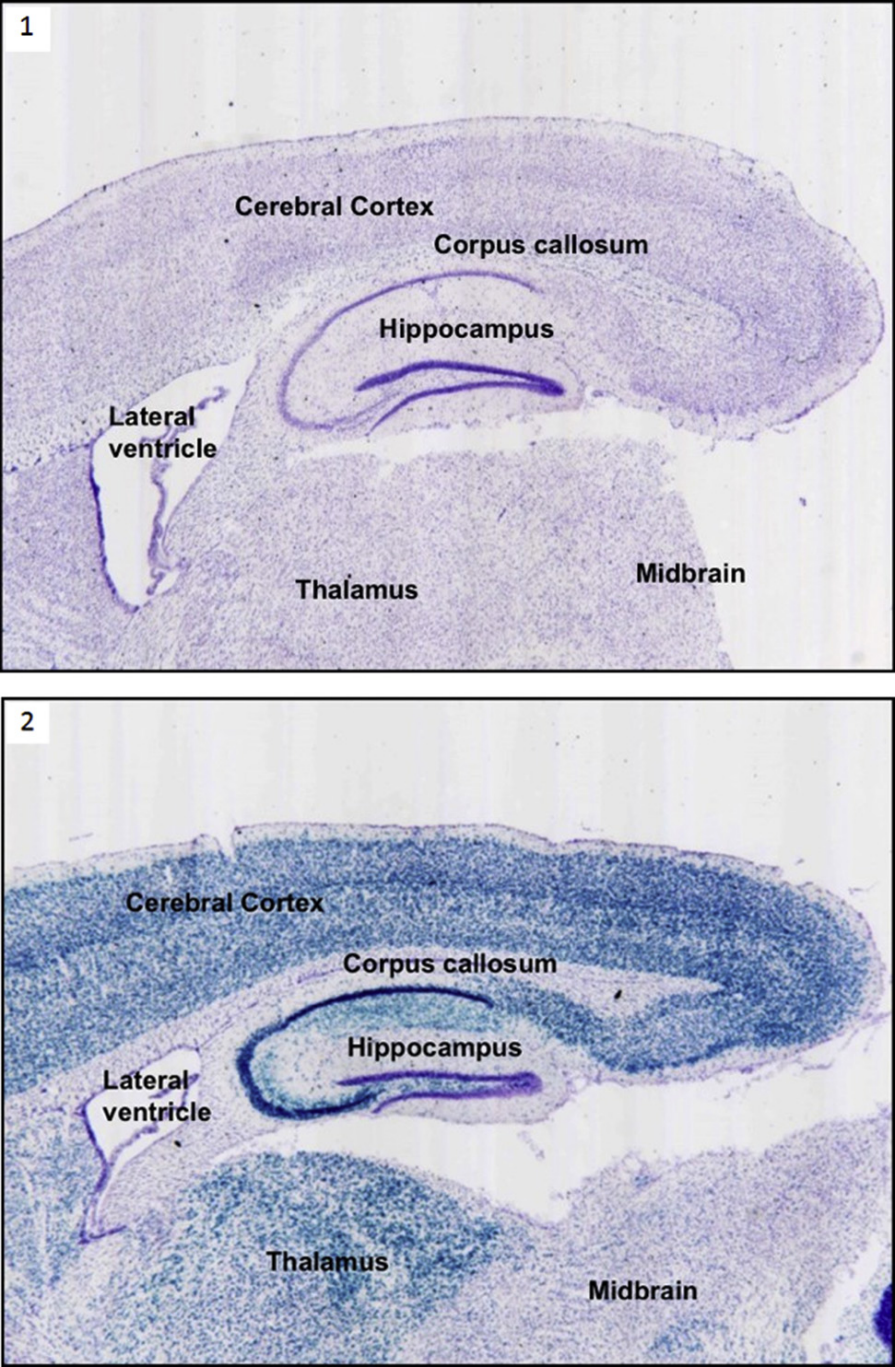
p110δ expression in the mouse brain. Brain sections of adult wild-type (−lacZ cassette) mice *(1)* and p110 d kinase dead (+lacZ cassette) β-gal reporter mice16 *(2)* stained with the neuronal stain cresyl violet *(purple)* and X-gal *(blue)* representing p110δ expression. Strong expression of p110δ was observed in areas of the hippocampus, cerebral cortex, and thalamus.

Global developmental or isolated speech delay were diagnosed against standard criteria by specialist pediatric services in 10 (19%) patients. Three further patients were treated for anxiety disorders, 1 with a diagnosis of autism, and 3 children were reviewed by psychological services for behavioral issues. Of note, PI3Kδ is strongly expressed in the mature and developing murine central nervous system (CNS; Fig 3).^16^

Individual patients were born with macrocrania, unilateral hypoplastic kidney, and unilateral microphthalmia.

### Thoracic radiology

Air-space opacity (Fig 4.1) was identified in 13 of 31 CT scans reviewed, and tree-in-bud opacities, bronchial wall thickening, or both were identified in 20 of 31 CT scans. Mosaic attenuation was present in 28 of 31 patients and classified as mild in 17, moderate in 7, and severe in 4 (Fig 4.2). Bronchiectasis was present in 21 of 31 scans, with an average of 3 lobes affected, and associated with atelectasis or lobar collapse in 12 patients. Sixteen patients had mediastinal lymphadenopathy, which was in a regional draining station to concurrent lobar consolidation in 4 instances. Follow-up imaging was available in 8 patients at a mean interval of 2.2 years. Four of the patients with air-space opacity, and regional lymphadenopathy showed resolution of presumed pneumonic changes but persistent volume loss, atelectasis, and development of bronchiectasis (Fig 4.1).

### Immunology laboratory results

**Fig 5:**
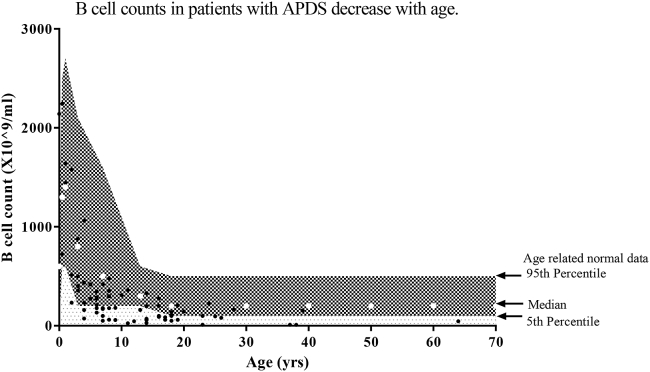
Age-related changes in B-cell counts in patients with APDS. Age-related median B-cell count *(white dots)*, B-cell count 5th to 95th percentile normal range *(checked area)*, and less than 5th percentile normal B-cell count *(spotted area)* were plotted.^12^

Lymphocyte immunophenotyping findings are summarized in Table II. Typical findings were reduced CD4 T-cell counts, increased CD8 T-cell counts of an effector/effector memory phenotype, and an expansion of transitional B cells. A history of herpesvirus infection was not associated with a deficiency in natural killer cells (*P* = .48), T_H_ cells (*P* = .47), or cytotoxic T cells (*P* = .35). Serial B-cell counts (n = 19) suggest that patients' B-cell levels decrease more quickly over time than in age-matched control subjects (Fig 5).

Immunoglobulin levels (Table III) were variable, with 43% of patients having reduced total IgG levels. Fifty-eight percent of patients with normal IgG levels had IgG_2_ subclass deficiency, and 89% who underwent testing exhibited a poor response to PPV. Reduced IgA (50%) and increased IgM (79%) levels were common. Two patients initially had marginally reduced IgM levels (age, 2 and 6 years), which over time became high (27 g/L) or normal (0.63 g/L), respectively. In 4 cases high IgM levels normalized after commencement of immunoglobulin replacement. One patient had a low IgG level after previous normal readings. Four patients with normal IgG and IgA levels responded poorly to PPV and had a previous diagnosis of specific antibody deficiency.^17^

### Treatment

#### Anti-infection prophylaxis

Sixty-two percent of the cohort currently receive and an additional 9% previously received antibiotic prophylaxis. Six (11%) patients are taking antiviral and 3 (6%) are taking antifungal prophylaxis.

#### Immunoglobulin replacement

Long-term immunoglobulin replacement was administered to 87% of the cohort, with reported benefit (reduction of infection) in the majority. In 3 patients aged 14 to 23 years, immunoglobulin replacement was switched to antibiotic prophylaxis (patient preference). The 7 patients who did not receive immunoglobulin replacement therapy included the 5 patients identified by genotyping relatives of patients with APDS.

#### HSCT

Five (9%) patients aged 5 to 14 years have undergone HSCT with medium- or reduced-intensity conditioning with a median follow-up after HSCT of 4.2 years (range, 1-14 years). Three transplantations (unrelated donors, one with 1A and 1B allelic mismatch) were successful, with minimal graft-versus-host disease, restoration of normal growth, and resolution of infection and nonneoplastic lymphoproliferation; chimerism in these patients ranged from 35% to 100%. A fourth procedure was complicated by poor engraftment (25% donor chimerism), resulting in long-term immunoglobulin therapy after transplantation. A fifth patient, who underwent splenectomy before transplantation, died of sepsis 2 years after HSCT.

#### Immunosuppression

Thirty percent of the cohort underwent at least 1 course of immunosuppressive therapy for lymphoproliferative, autoimmune, or inflammatory disease. Rituximab was of benefit in the management of autoimmune hemolytic anemia (n = 8) and nonneoplastic lymphoproliferation (n = 5) although often complicated by sustained B-cell lymphopenia. Six patients were treated with rapamycin; 5 experienced benefit, with a decrease in nonneoplastic or neoplastic lymphoproliferation, but therapy was stopped in the fifth patient because of side effects.

### Fatal outcomes

Five patients with APDS died, 3 (aged 1, 19, and 27 years) from lymphoma, 1 (aged 14 years) from sepsis after splenectomy and HSCT, and 1 (aged 39 years) from respiratory failure and chronic lung infection. Additionally, infection-related deaths in childhood and early adult life (≤30 years old) were reported for 5 nongenotyped relatives of patients with APDS.

## Discussion

**Fig 6:**
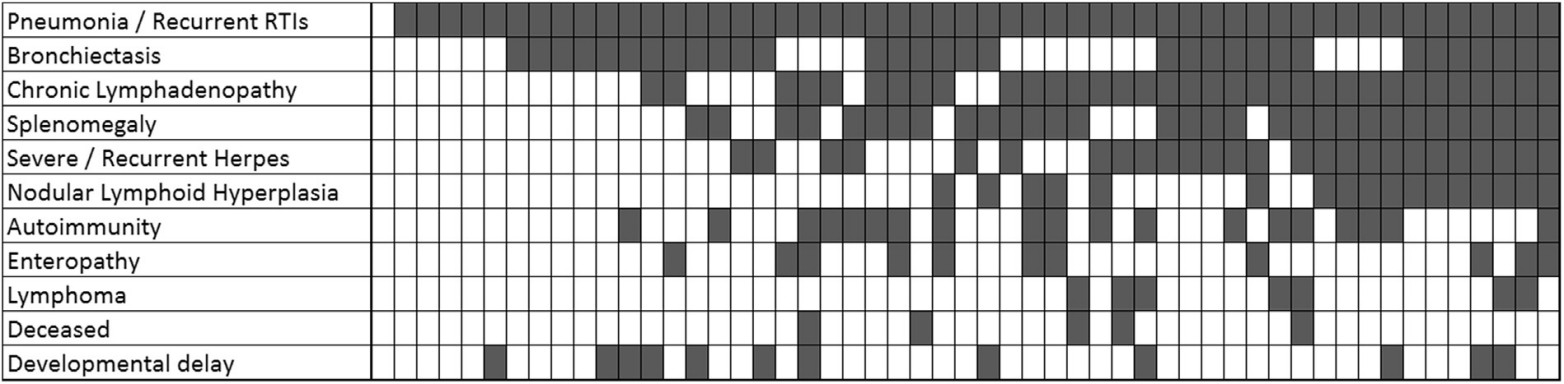
Variation in clinical phenotypes of APDS. Each *column* represents a patient with APDS. Each *row* represents a frequent or serious complication of APDS. *White boxes* and *gray boxes* depict the absence or presence of a complication, respectively.

We present an overview of the clinical course of APDS in the largest cohort to date with confirmed GOF *PIK3CD* mutations. The phenotype is highly variable (Fig 6), ranging from asymptomatic adults to profound immunodeficiency causing early death or necessitating HSCT in childhood; the clinical features overlap those of other primary immunodeficiencies, such as cytotoxic T lymphocyte–associated antigen 4 (CTLA4) and LPS-responsive beige-like anchor protein (LRBA) deficiency. Interestingly, 3 recent publications^18^, ^19^, ^20^ describe heterozygous mutations in the *PIK3R1* gene (encoding the PI3K regulatory subunit), leading to hyperactivation of PI3Kδ and a clinical syndrome (APDS2 or PASLI-R1) highly reminiscent of that described herein. Conversely, a recessive mutation in *PIK3R1*, resulting in loss of p85α expression, was reported in a patient with agammaglobulinemia and absent B-cell lineage.^21^ Together with the aberrant lymphocyte function in mice lacking PI3Kδ activity,^22^ these findings indicate that balanced signaling in the PI3Kδ pathway is critical for normal immune function.

Recurrent respiratory tract infection is almost universally found in patients with APDS. Bacterial isolates were typical for antibody deficiency, and the incidence of bronchiectasis was similar or higher than in previously described common variable immune deficiency cohorts (see Table E1 in this article's Online Repository at www.jacionline.org).^23^, ^24^, ^25^, ^26^ Notably, 63% (20/32) of patients with bronchiectasis had normal total IgG levels, suggesting that patients with early-onset bronchiectasis and even minor immunoglobulin abnormalities should be screened for APDS mutations. Increased IgM levels were seen in 82% of the cohort, reminiscent of a class-switch recombination defect.^7^, ^8^ Thus we propose that patients presenting with reduced IgG and IgA levels and normal or increased IgM levels,^17^ particularly those with normal CD40 ligand expression, should be screened for activating PI3Kδ mutations.

Almost half of our cohort had difficulty in resolving herpesvirus infections, particularly EBV and CMV. There was no association between herpesvirus infections and decreased T_H_, cytotoxic T-cell, or natural killer cell counts, suggesting a functional defect underlies this susceptibility. Diffuse lymphadenopathy was associated with systemic herpes infections, with consistent features on lymph node histology. Other opportunistic infections were uncommon, and patients did not experience *Pneumocystis jirovecii* pneumonia. *Cryptosporidium* species was identified in only 2 cases, one of whom had cholangitis and liver disease, which is normally associated with MHC class II or IL-21/IL-21 receptor deficiencies but also described in CD40 ligand and CD40 deficiency.^27^ Persistent granulomatous skin lesions after BCG vaccination occurred in 2 patients, but no other mycobacterial infections were reported. Although there was a moderate excess of skin infections and abscesses, there were no cases of invasive staphylococcal or *Aspergillus* species infections to suggest major neutrophil dysfunction.

Although APDS can present as a common variable immune deficiency–like disease, it is also characterized by viral infections; lymphocyte immunophenotyping confirms APDS is a combined immunodeficiency. The typical T-cell profile was of reduced T_H_ cells and recent thymic emigrants, whereas cytotoxic T cells had a predominantly effector or activated phenotype. B-cell numbers were often normal in early life but decreased with time. The reduction in B-cell counts, including class-switched memory B cells and expansion of transitional B cells, suggests defects in B-cell maturation or enhanced mature B-cell death.^22^

The development of focal bronchiectasis observed after consolidative changes strengthens the suspected causal link between infection and airway damage. Consistent with a role for infection in the florid nonneoplastic lymphoproliferation characteristic of patients with APDS, lymphadenopathy was often associated with regional (mediastinal lymphadenopathy in bronchiectatic patients) or systemic infection (herpesviral infections) and tended to improve on infection resolution. Our review of chest CT scans also revealed an unexpectedly high incidence (28/31) of mosaic attenuation, which is indicative of reduced perfusion of poorly ventilated lung regions. This might reflect inflammatory small-airway disease or result from viral respiratory tract infections.

Patients with APDS had a high incidence (34%) and wide range of inflammatory/autoimmune manifestations. Enhanced PI3Kδ activity has been reported in patients with autoimmune diseases, such as systemic lupus erythematosus,^28^ and PI3Kδ modulates regulatory T-cell function.^29^ Our findings suggest a role for PI3Kδ in the genesis or perpetuation of autoimmunity and potentially for PI3Kδ inhibition in treating such conditions. Activating somatic *PIK3CD* mutations have been associated with lymphoid malignancy.^30^ We identified 7 lymphomas in this series of 53 patients with a spectrum of pathologic subtypes but identified no solid malignancies, perhaps reflecting the young age of our cohort or the predominant expression of p110δ in leukocytes. Although PI3Kδ is described as leukocyte restricted, expression is also found in cells of breast or melanocytic origin,^31^ lung fibroblasts,^32^ and TNF-α–stimulated endothelial and synovial cells.^33^ p110δ has recently been shown to regulate epithelial cell polarity,^34^ which is of potential import for respiratory epithelial function. It is tempting to speculate that induction of p110δ expression by locally produced TNF-α during inflammation might impair epithelial barrier functions and aggravate local inflammation. Thus the lung phenotype might be the result of interplay between immune functions of p110δ and epithelium-intrinsic roles of p110δ.

Almost one fifth of our cohort experienced neurodevelopmental morbidity, from speech delay to global developmental delay. PI3Kδ is expressed broadly in the developing CNS, as well as in specific adult brain regions (including the hippocampus, cerebral cortex, and thalamus) of reporter mice (Fig 3).^16^ PI3Kδ has been implicated in schizophrenia; pharmacologic inhibition reversed prepulse inhibition deficits in a rat model of schizophrenia and blocked amphetamine-induced hyperlocomotion in a mouse model of psychosis-like behavior.^35^ Interestingly, loss-off-function phosphatase and tensin homolog *(PTEN)* mutations (with consequent enhanced PI3K-dependent signaling) are associated macrocrania and autism spectrum disorders.^36^ One patient with APDS had macrocrania, and in addition to the single patient with a formal diagnosis of autism in our cohort, before submission of this manuscript, we were informed of an additional patient with APDS with autism spectrum disorder (personal communication; Professor P. Martin von Hagen, Erasmus MC, The Netherlands). These findings suggest PI3Kδ might play an important but little-understood role in the CNS, and this aspect of APDS warrants further study.

HSCT has been seemingly curative in 3 patients with APDS described herein and an additional 5 patients described by Imai et al,^37^ supporting its use in carefully selected cases; however, longer-term follow-up to determine the degree of donor chimerism needed to achieve cure is required. Lucas et al^2^ reported a single patient in whom the mammalian/mechanistic target of rapamycin inhibitor rapamycin improved circulating T-cell profiles. Four patients within our cohort experienced a decrease in nonneoplastic lymphoproliferation while taking rapamycin, and this drug also led to regression of cutaneous T-cell lymphoma. Nevertheless, direct inhibition of activated PI3Kδ might be a more attractive approach in patients with APDS. Selective PI3Kδ inhibitors are currently in clinical trials for a range of cancers and inflammatory disorders, and one compound is already approved for treatment of B-cell malignancies.^38^, ^39^ Such disease-specific therapy could address both the infectious and noninfectious complications of APDS, but the reported side effect profile and significant immunoparesis in mice lacking PI3Kδ function^25^ emphasize the need for careful dosing to restore normal rather than abolish PI3Kδ activity, particularly given that long-term treatment is contemplated.

In conclusion, APDS is a combined immune deficiency with a variable phenotype complicated by recurrent sinopulmonary bacterial and herpesvirus infections, bronchiectasis, lymphoid hyperplasia, autoimmunity, and, less frequently, neurodevelopmental delay and lymphoma. The rapidly increasing number of patients identified since the initial description of APDS in 2013 suggests this is a clinically significant cause of primary immunodeficiencies, which should be considered in patients presenting with atypical or inherited primary antibody deficiency, bronchiectasis, severe herpesvirus infections, and lymphoma. The severity of complications and significant mortality rate support the consideration of HSCT in young patients, as well as clinical trials of selective PI3Kδ inhibitors for this condition.

**Clinical implications**

The variable clinical phenotype with severe complications of bronchiectasis, bacterial and viral infections, and lymphoma suggests that patients who fit this clinical profile should be screened for APDS-causing mutations.

## Figures and Tables

**Table E1:** Comparison of the frequency of complications in patients with APDS and common variable immune deficiency

Clinical feature	Frequency (%) in APDS cohort	Frequency (%) in CVID cohort
Pneumonia	85	32-77E1, E2, E3, E4
Bronchiectasis	60	23-64E1, E3, E5, E6, E7
Splenomegaly	58	15-30E1, E3, E4, E5, E6
Autoimmunity	42	22-29E1, E2, E3
Enteropathy	25	9E1, E4, E5
Granuloma∗	0	8-9E1, E2, E5
Meningitis/encephalitis	1.9	3-4E1, E4
Lymphoma	11	3-8E1, E2, E5
Living patients currently receiving immunoglobulin replacement therapy	77	80^E1^

*CVID*, Common variable immune deficiency.

∗ Two patients with cutaneous granulomatous inflammation after BCG vaccination were not included.

**Fig 4:**
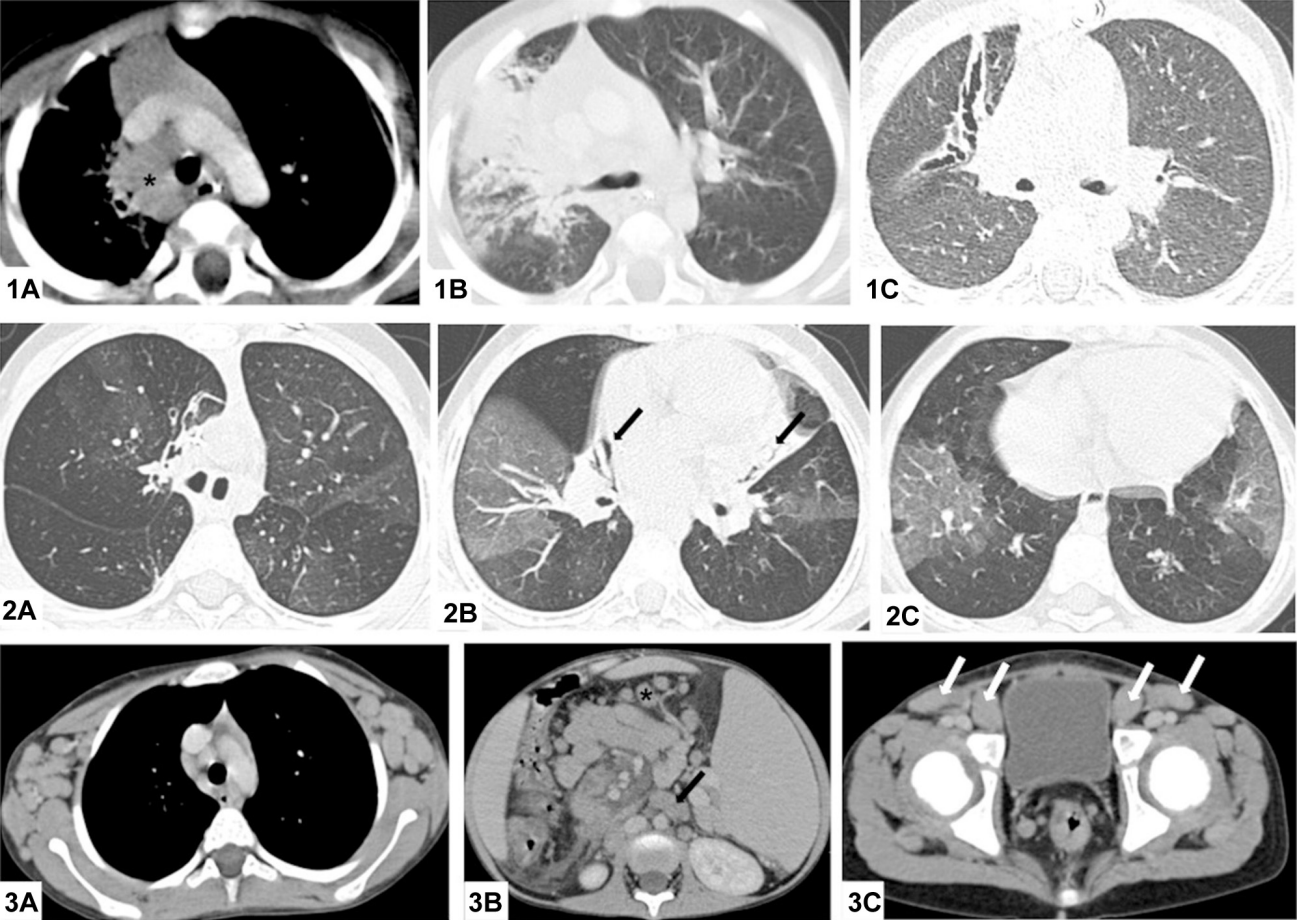
Radiology of patients with APDS. *1*, CT scan of the chest (2-year-old boy), demonstrating right paratracheal lymphadenopathy *(A)*, right upper lobe consolidation, and centrilobular nodules *(B)*, progressing 2 years later to severe right upper lobe bronchiectasis *(C)*. *2*, CT scan of the chest *(A-C)* in a 7-year-old boy reveals widespread mosaic attenuation (indicative of small airways disease), mild right upper lobe bronchiectasis *(A)* and atelectasis (*B*, *black arrows*). *3*, CT scan of the chest *(A)*, abdomen *(B)*, and pelvis *(C)* of an 8-year-old boy showing axillary, paratracheal, para-aortic *(black arrow)*, mesenteric and inguinal lymphadenopathy *(white arrows)*, and splenomegaly.

**Table I:** Clinical manifestations of APDS

	Frequency, n/total studied (%)
Infectious complication	
Recurrent respiratory tract infections	51/53 (98)
Pneumonia†	39/46 (85)∗
Bronchiectasis‡	32/53 (60)
Chronic rhinosinusitis	24/53 (45)
Recurrent otitis media (with permanent hearing loss)	26/53 (49) 4/53 (8)
Severe or persistent herpesvirus infection	26/53 (49)
EBV	14/53 (26)
CMV	8/53 (15)
HSV and VZV	11/53 (21)
Tonsillitis (with tonsillectomy)	15/53 (28) 7/53 (13)
Ocular infections	10/53 (19)
Noninfectious complication	
Lymphadenopathy§	34/53 (64)
Splenomegaly	31/53 (58)
Hepatomegaly	24/53 (45)
Autoimmune disease	22/53 (42)
Nodular mucosal lymphoid hyperplasia	17/53 (32)
Enteropathy‖	13/53 (25)
Developmental delay	10/53 (19)
Lymphoma	7/53 (13)

Total studied = 53 unless otherwise indicated.

*VZV*, Varicella zoster virus.

∗ N = 46 because 7 patients had no chest radiology available.

† Pneumonia was defined as at least 1 clinically and radiologically diagnosed pneumonia episode.

‡ Bronchiectasis diagnosed on thoracic CT imaging.

§ Lymphadenopathy persistent for at least 3 months.

‖ Nine of 13 patients with enteropathy had gastrointestinal nodular mucosal lymphoid hyperplasia confirmed on endoscopy.

**Table II:** Summary of lymphocyte phenotypic characteristics of APDS

Lymphocyte subpopulation∗	Frequency, n/total studied (%)
T cells	
Reduced T_H_ cell counts (CD3^+^CD4^+^)	43/51 (84)
Reduced recent thymic emigrant T-cell counts (CD3^+^CD4^+^CD45RA^+^CD31^+^)	14/22 (64)
Normal cytotoxic T-cell counts (CD3^+^CD8^+^)	34/51 (67)
Reduced cytotoxic T-cell counts (CD3^+^CD8^+^)	14/51 (27)
Increased effector-effector memory cytotoxic T-cell counts (CD3^+^CD8^+^CCR7^−^CD45RA^+/−^)	17/18 (94)
Reversed CD4/CD8 ratio	33/51 (65)
B cells	
Reduced B-cell counts (CD19^+^)	32/48 (67)
Increased transitional B-cell counts (CD19^+^IgM^++^CD38^++^)	24/32 (75)
Reduced nonswitched memory B cells (CD19^+^IgD^+^CD27^+^)	15/30 (50)
Reduced class-switched memory B-cell counts (CD19^+^IgD^−^CD27^+^)	17/30 (57)
NK cells	
Normal NK cell counts (CD16^+^CD56^+^)	28/43 (65)
Reduced NK cell counts (CD16^+^CD56^+^)	12/43 (28)

*NK*, Natural killer.

∗ Results were deemed reduced, normal, or increased with reference to age-related normal ranges.^12^, ^13^, ^14^ Most recent results available were used, and B-cell levels after rituximab were excluded.

**Table III:** Summary of immunoglobulin characteristics of the APDS cohort

	Reduced, n/total (%)	Normal, n/total (%)	Increased, n/total (%)
IgG	21/49 (43)	26/49 (53)	2/49 (4)
IgA	25/50 (50)	24/50 (48)	1/50 (0.5)
IgM	0/50 (0)	12/50 (24)	38/50 (76)
Pneumococcal vaccine response∗	25/28 (89)	3/28 (11)	

Immunoglobulin results were deemed reduced, normal, or increased with reference to age-related normal ranges.^15^

∗ A poor pneumococcal polysaccharide vaccine response was defined as less than 4-fold increase in anti-pneumococcal IgG titer at 4 to 6 weeks after PPV vaccination. Of the 25 patients in whom pneumococcal responses were not available, 15 had reduced IgG levels and received immunoglobulin replacement therapy.

## References

[1] Angulo I., Vadas O., Garçon F., Banham-Hall E., Plagnol V., Leahy T.R. (2013). Phosphoinositide 3-kinase δ gene mutation predisposes to infection and airway damage. Science.

[2] Lucas C.L., Kuehn H.S., Zhao F., Niemela J.E., Deenick E.K., Palendira U. (2014). Dominant-activating germline mutations in the gene encoding the PI(3)K catalytic subunit p110δ result in T cell senescence and human immunodeficiency. Nat Immunol.

[3] Vanhaesebroeck B., Welham M.J., Kotani K., Stein R., Warne P.H., Zvelebil M.J. (1997). p110δ, a novel phosphoinositide 3-kinase in leukocytes. Proc Natl Acad Sci U S A.

[4] Chantry D., Vojtek A., Kashishian A., Holtzman D.A., Wood C., Gray P.W. (1997). p110δ, a novel phosphatidylinositol 3-kinase catalytic subunit that associates with p85 and is expressed predominantly in leukocytes. J Biol Chem.

[5] Kok K., Geering B., Vanhaesebroeck B. (2009). Regulation of phosphoinositide 3-kinase expression in health and disease. Trends Biochem Sci.

[6] Jou S.T., Chien Y.H., Yang Y.H., Wang T.C., Shyur S.D., Chou C.C. (2006). Identification of variations in the human phosphoinositide 3-kinase p110delta gene in children with primary B-cell immunodeficiency of unknown aetiology. Int J Immunogenet.

[7] Kracker S., Curtis J., Ibrahim M.A.A., Sediva A., Salisbury S., Campr V. (2014). Occurrence of B-cell lymphomas in patients with Activated Phosphoinositide 3-Kinase δ syndrome (APDS). J Allergy Clin Immunol.

[8] Crank M.C., Grossman J.K., Moir S., Pittaluga S., Buckner C.M., Kardava L. (2014). Mutations in PIK3CD can cause hyper IgM syndrome (HIGM) associated with increased cancer susceptibility. J Clin Immunol.

[9] Hartman H.N., Niemela J., Hintermeyer M.K., Garofalo M., Stoddard J., Verbsky J.W. (2015). Gain of function mutations in PIK3CD as a cause of primary sclerosing cholangitis. J Clin Immunol.

[10] Hansell D.M., Bankier A.A., MacMahon H., McLoud T.C., Müller N.L., Remy J. (2008). Fleischner Society: glossary of terms for thoracic imaging. Radiology.

[11] Copley S.J., Wells A.U., Müller N.L., Rubens M.B., Hollings N.P., Cleverley J.R. (2002). Thin-section CT in obstructive pulmonary disease: discriminatory value. Radiology.

[12] Comans-Bitter W.M., de Groot R., van den Beemd R., Neijens H.J., Hop W.C., Groeneveld K. (1997). Immunophenotyping of blood lymphocytes in childhood. Reference values for lymphocyte subpopulations. J Pediatr.

[13] Piątosa B., Wolska-Kuśnierz B., Pac M., Siewiera K., Gałkowska E., Bernatowska E. (2010). B cell subsets in healthy children: reference values for evaluation of B cell maturation process in peripheral blood. Cytometry B Clin Cytom.

[14] Morbach H., Eichhorn E.M., Liese J.G., Girschick H.J. (2010). Reference values for B cell subpopulations from infancy to adulthood. Clin Exp Immunol.

[15] Sheffield Protein Reference Unit. Available at: www.immqas.org.uk/pru.asp?ID=316. Accessed June 27, 2016.

[16] Eickholt B.J., Ahmed A.I., Davies M., Papakonstanti E.A., Pearce W., Starkey M.L. (2007). Control of axonal growth and regeneration of sensory neurons by the p110delta PI 3-kinase. PLoS One.

[17] Al-Herz W., Bousfiha A., Casanova J.L., Chatila T., Conley M.E., Cunningham-Rundles C. (2014). Primary immunodeficiency diseases: an update on the classification from the international union of immunological societies expert committee for primary immunodeficiency. Front Immunol.

[18] Deau M.C., Heurtier L., Frange P., Suarez F., Bole-Feysot C., Nitschke P. (2014). A human immunodeficiency caused by mutations in the PIK3R1 gene. J Clin Invest.

[19] Lucas C.L., Zhang Y., Venida A., Wang Y., Hughes J., McElwee J. (2014). Heterozygous splice mutation in *PIK3R1* causes human immunodeficiency with lymphoproliferation due to dominant activation of PI3K. J Exp Med.

[20] Elkaim E., Neven B., Bruneau J., Mitsui-Sekinaka K., Stanislas A., Heurtier L. (2016). Clinical and immunological phenotype associated with activated PI3k-delta syndrome 2 (APDS2/PASLI-R1)—a cohort study. J Allergy Clin Immunol.

[21] Conley M.E., Dobbs A.K., Quintana A.M., Bosompem A., Wang Y.D., Coustan-Smith E. (2012). Agammaglobulinaemia and absent B lineage cells in a patient lacking the p85α subunit of PI3K. J Exp Med.

[22] Okkenhaug K., Bilancio A., Farjot G., Priddle H., Sancho S., Peskett E. (2002). Impaired B and T cell antigen receptor signaling in p110delta PI 3-kinase mutant mice. Science.

[23] Gathmann B., Mahlaoui N., Gérard L., Oksenhendler E., Warnatz K., Schulze I., CEREDIH (2014). Clinical picture and treatment of 2212 patients with common variable immunodeficiency. J Allergy Clin Immunol.

[24] Chapel H., Lucas M., Lee M., Bjorkander J., Webster D., Grimbacher B. (2008). Common variable immunodeficiency disorders: division into distinct clinical phenotypes. Blood.

[25] Thickett K.M., Kumararatne D.S., Banerjee A.K., Dudley R., Stableforth D.E. (2002). Common variable immune deficiency: respiratory manifestations, pulmonary function and high-resolution CT scan findings. QJM.

[26] Quinti I., Soresina A., Spadaro G., Martino S., Donnanno S., Agostini C. (2007). Long-term follow-up and outcome of a large cohort of patients with common variable immunodeficiency. J Clin Immunol.

[27] Winkelstein J.A., Marino M.C., Ochs H., Fuleihan R., Scholl P.R., Geha R. (2003). The X-linked Hyper-IgM Syndrome: clinical and immunologic features of 79 patients. Medicine (Baltimore).

[28] Suárez-Fueyo A., Barber D.F., Martínez-Ara J., Zea-Mendoza A.C., Carrera A.C. (2011). Phosphoinositide 3-kinase delta activity is a frequent event in systemic lupus erythematosus that confers resistance to activation-induced T cell death. J Immunol.

[29] Patton D.T., Garden O.A., Pearce W.P., Clough L.E., Monk C.R., Leung E. (2006). Cutting edge: the phosphoinositide 3-kinase p110 delta is critical for the function of CD4+CD25+Foxp3+ regulatory T cells. J Immunol.

[30] Zhang J., Grubor V., Love C.L., Banerjee A., Richards K.L., Mieczkowski P.A. (2013). Genetic heterogeneity of diffuse large B-cell lymphoma. Proc Natl Acad Sci U S A.

[31] Sawyer C., Sturge J., Bennett D.C., O'Hare M.J., Allen W.E., Bain J. (2003). Regulation of breast cancer cell chemotaxis by the phosphoinositide 3-kinase p110delta. Cancer Res.

[32] Conte E., Fruciano M., Fagone E., Gili E., Caraci F., Iemmolo M. (2011). Inhibition of PI3K prevents the proliferation and differentiation of human lung fibroblasts into myofibroblasts: the role of class 1 isoforms. PLoS One.

[33] Whitehead M.A., Bombardieri M., Pitzalis C., Vanhaesebroeck B. (2012). Isoform induction of human p110d PI3K expression by TNFalpha: identification of a new and inducible PIK3CD promoter. Biochem J.

[34] Peng J., Awad A., Sar S., Hamze Komaiha O., Moyano R., Rayal A. (2015). Phosphoinositide 3-kinase p110δ promotes lumen formation through the enhancement of apico-basal polarity and basal membrane organization. Nat Commun.

[35] Law A.J., Wang Y., Sei Y., O'Donnell P., Piantadosi P., Papaleo F. (2012). Neuregulin 1-ErbB4-PI3K signaling in schizophrenia and phosphoinositide 3-kinase-p110δ inhibition as a potential therapeutic strategy. Proc Natl Acad Sci U S A.

[36] Spinelli L., Black F.M., Berg J.N., Eickholt B.J., Leslie N.R. (2015). Functionally distinct groups of inherited PTEN mutations in autism and tumour syndromes. J Med Genet.

[37] Imai K., Tsujita Y., Mitsui-Sekinaka K., Mitsuiki N., Takashima T., Okano T. (2014). Hematopoietic stem cell transplantation for the patients with activated PI3K-delta syndrome. J Clin Immunol.

[38] Furman R.R., Sharman J.P., Coutre S.E., Cheson B.D., Pagel J.M., Hillmen P. (2014). Idelalisib and rituximab in relapsed chronic lymphocytic leukemia. N Engl J Med.

[39] Gopal A.K., Kahl B.S., de Vos S., Wagner-Johnston N.D., Schuster S.J., Jurczak W.J. (2014). PI3Kδ inhibition by idelalisib in patients with relapsed indolent lymphoma. N Engl J Med.

